# Investigation of Mutated in Colorectal Cancer (MCC) Gene Family Evolution History Indicates a Putative Role in Th17/Treg Differentiation

**DOI:** 10.3390/ijms241511940

**Published:** 2023-07-26

**Authors:** Norwin Kubick, Justyna Paszkiewicz, Irmina Bieńkowska, Michał Ławiński, Jarosław Olav Horbańczuk, Mariusz Sacharczuk, Michel Edwar Mickael

**Affiliations:** 1 Department of Biology, Institute of Plant Science and Microbiology, Univeristy of Hamburg, Ohnhorststr. 18, 22609 Hamburg, Germany; n.kubick@uke.de; 2 Department of Health, John Paul II University of Applied Sciences in Biala Podlaska, Sidorska 95/97, 21-500 Biała Podlaska, Poland; j.paszkiewicz@dyd.akademiabialska.pl; 3 Institute of Animal Biotechnology and Genetics, Polish Academy of Science, Postępu 36A, 05-552 Jastrzębiec, Poland; i.bienkowska@igbzpan.pl (I.B.); michal-lawinski@wp.pl (M.Ł.); j.horbanczuk@igbzpan.pl (J.O.H.); 4 Department of General Surgery, Gastroenterology and Oncology, Medical University of Warsaw, 02-091 Warsaw, Poland; 5 Department of Pharmacodynamics, Faculty of Pharmacy, Medical University of Warsaw, l Banacha 1, 02-697 Warsaw, Poland; 6 PM Research Center, Väpnaregatan 22, 58649 Linköping, Sweden

**Keywords:** Th17, Treg, differentiation, colorectal cancer, evolution

## Abstract

The MCC family of genes plays a role in colorectal cancer development through various immunological pathways, including the Th17/Treg axis. We have previously shown that MCC1 but not MCC2 plays a role in Treg differentiation. Our understanding of the genetic divergence patterns and evolutionary history of the MCC family in relation to its function, in general, and the Th17/Treg axis, in particular, remains incomplete. In this investigation, we explored 12 species’ genomes to study the phylogenetic origin, structure, and functional specificity of this family. In vertebrates, both MCC1 and MCC2 homologs have been discovered, while invertebrates have a single MCC homolog. We found MCC homologs as early as Cnidarians and Trichoplax, suggesting that the MCC family first appeared 741 million years ago (Ma), whereas MCC divergence into the MCC1 and MCC2 families occurred at 540 Ma. In general, we did not detect significant positive selection regulating MCC evolution. Our investigation, based on MCC1 structural similarity, suggests that they may play a role in the evolutionary changes in Tregs’ emergence towards complexity, including the ability to utilize calcium for differentiation through the use of the EFH calcium-binding domain. We also found that the motif NPSTGE was highly conserved in MCC1, but not in MCC2. The NPSTGE motif binds KEAP1 with high affinity, suggesting an Nrf2-mediated function for MCC1. In the case of MCC2, we found that the “modifier of rudimentary” motif is highly conserved. This motif contributes to the regulation of alternative splicing. Overall, our study sheds light on how the evolution of the MCC family is connected to its function in regulating the Th17/Treg axis.

## 1. Introduction

The Th17/Treg axis is crucial for both promoting and repressing colorectal cancer. Th17 and Treg belong to the CD4+ T cell population [1], and while they share a large portion of their transcriptome [2], they have functionally diverged. Th17 cells can be proinflammatory by producing several cytokines, such as IL17A, IL17F, IL1, and IL6 [3]. It has been shown that Th17 cells stimulate the infiltration of cytotoxic CD8+ T lymphocytes into colorectal cancer tissues, thus supporting the body in the fight against cancer [4]. In contrast to Th17, Treg cells support colorectal cancer growth [5,6]. Treg cells are also known to be able to inhibit Th17 cells through both direct and indirect pathways.

MCC/MCC1 (“mutated in colorectal cancer”) plays a significant role in colorectal cancer progression. MCC1 was found to be linked to the familial adenomatous polyposis susceptibility locus on chromosome 5q. Familial adenomatous polyposis is a colorectal cancer risk factor because hereditary precancerous colorectal polyps can evolve into colorectal cancer [7]. Recently, it was shown that MCC1 could be contributing to cancer progression through the dysregulation of the WNT pathway [8]. Additionally, recent research has revealed that the nuclear factor-B (NF-κB) pathway and cell cycle regulation, two crucial cellular processes related to carcinogenesis, may be affected by MCC1 expression [9]. MCC1 belongs to the MCC family, which includes another homolog known as MCC2/USHBP1 (Usher syndrome type 1C Harmonin-binding protein 1). However, the function of MCC2 is still unclear [10]. Previously, we found, upon an inspection of microarray/RNAseq data that compare Th17 and Treg differentiation, that MCC1 and not MCC2 is upregulated in Treg, but not in Th17, along with a host of genes that are associated with cell cycle regulation [11]. However, the phylogenetic relationship between the two proteins is currently unknown [11].

In this report, we conducted an extensive phylogenetic analysis of the MCC family to investigate its role in the context of the Th17/Treg axis function in colorectal cancer. We found a single MCC homolog in various invertebrate species, including Spirlai, Arthopoda, Cnidria, and Trichoplx. During the Cambrian explosion (e.g., during Vertebrate emergence) and the two rounds of genome duplication (2R), two homologs, namely MCC1 and MCC2, emerged. The main building blocks of MCC1 in vertebrates are two domains of MCC-PDZ and a single EF-hand domain. This structure is also found in Oedothorax gibbosus (Gibbous dwarf spider) and is partially conserved in other investigated invertebrate species, hinting toward the functional conservation between MCC and MCC1. MCC2 sequences seem to have lost EF-hand domains. Our investigation indicates that the nearest homolog to the MCC family ancestral sequence homolog is a protein containing an EF-hand domain. Moreover, motif inspection suggests that the MCC1 and MCC2 families could be playing a primary role in cell cycle regulation. Additionally, we found that the motif NPSTGE, which is known to play a role in its interaction with KEAP1, is conserved in MCC1. Our findings suggest that MCC1 could be enhancing Treg differentiation by inhibiting the KEAP1 effect.

## 2. Results

### 2.1. Phylogenetic Analysis

**Figure 1:**
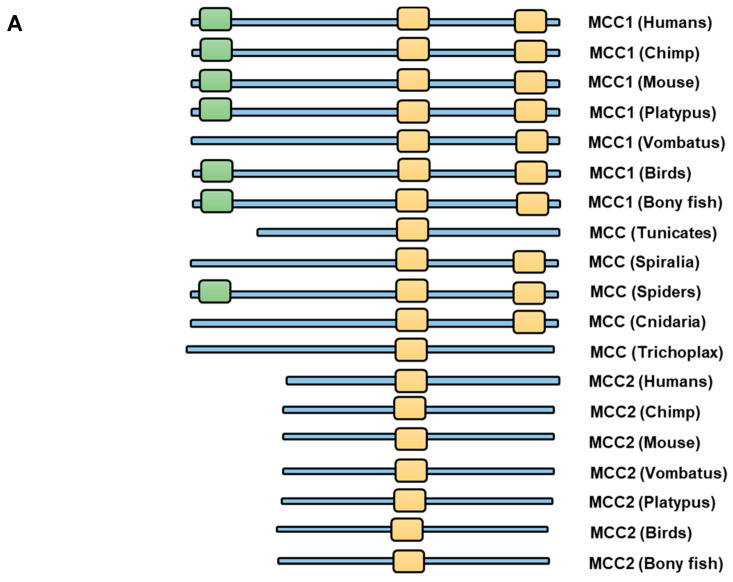

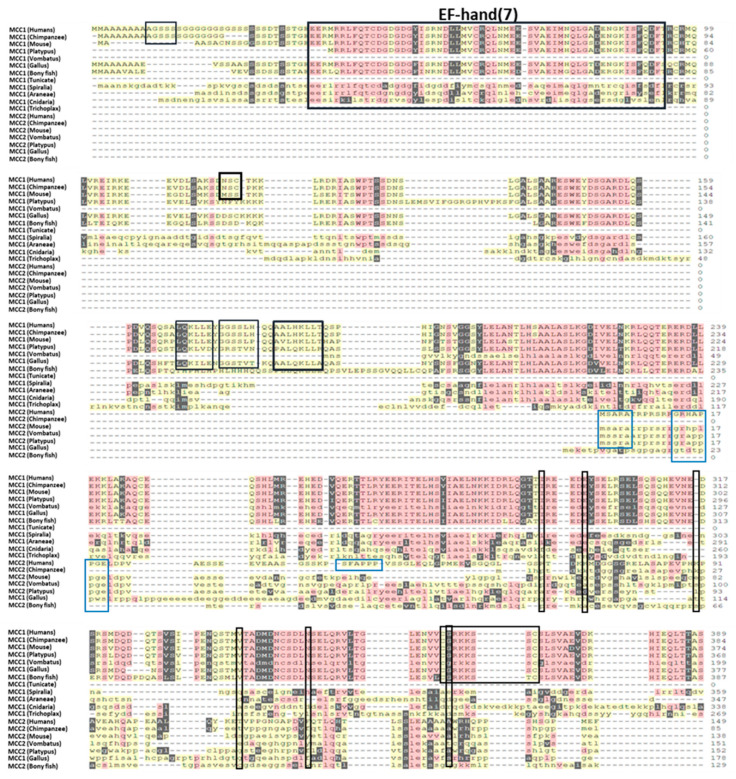

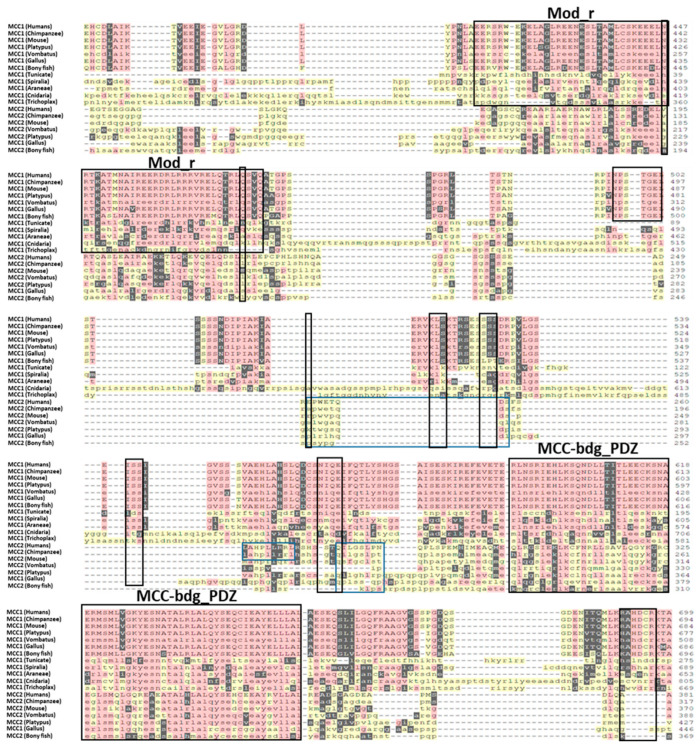

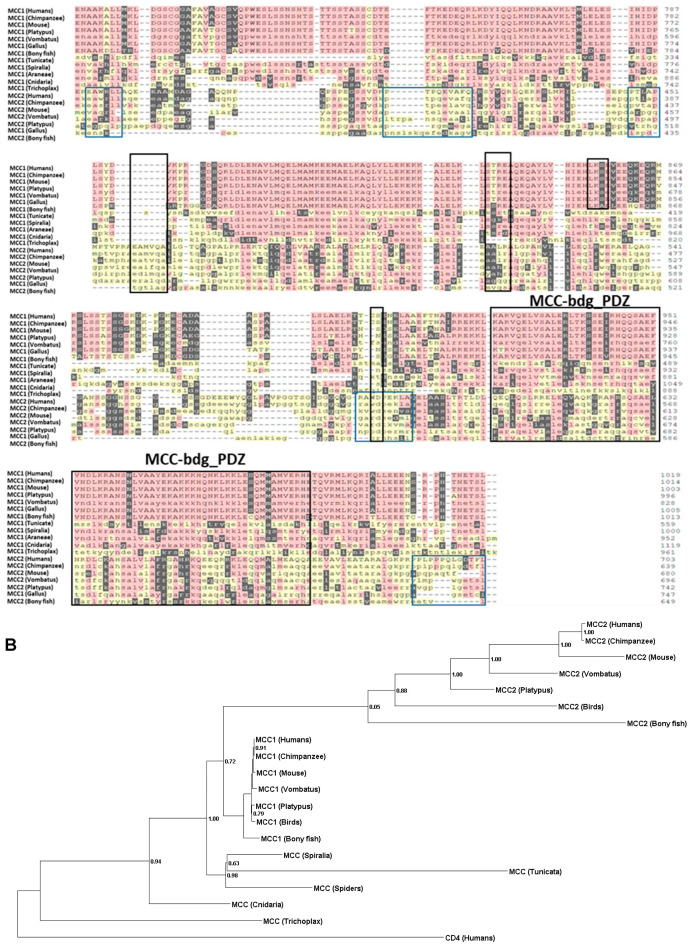
Phylogenetic analysis of the MCC family. (**A**) Multiple sequence alignment of the MCC family members show a high degree of homology. EFHAND(7) is shown in light red, and the first and second MCC-BDG-PDZ are shown in light orange. (**B**) The two rounds of duplication are clear within the family species. Only one homolog for the MCC family appears in invertebrates. During the Cumbrian explosion of vertebrates, gene duplication occurred, giving rise to two distinct homologs: MCC1 and MCC2.

Phylogenetic analysis indicates that two rounds of duplications resulted in the divergence of MCC1 and MCC2. We downloaded human MCC1 and MCC2 protein sequences from the GEO protein repository. We utilized the Blastp server to acquire homologous proteins for MCC1 and MCC2 among twelve species. We employed Seaview to perform the multiple sequence alignment using the MUSCLE algorithm (Figure 1A). After that, we constructed the phylogenetic tree using the PyML method utilizing the LG model (Figure 1B). We found a single homolog of the MCC in invertebrates. Our research identified a putative MCC homolog (e.g., XP_019849192.1) in *Amphimedon queenslandica*. However, Blastp’s E-value was lower than our threshold of 1 × 10^−10^. Thus, that sequence was not accepted. Two MCC homologs were found in all vertebrate species investigated. The bony fish seems to be the first vertebrate to possess a pair of MCC homologs. These results indicate that the divergence of the MCC1 and MCC2 homologs occurred at the 2R stage during the Cumbrian explosion. Our findings indicate that all vertebrate MCC1 sequences and MCC sequences in Cnidaria, Spiralia, and Arthropoda possess two MCC-PDZ domains. In contrast, all MCC2 sequences, as well as Tunicate and Trichoplax MCC sequences, feature only a single MCC-PDZ domain. It is noteworthy that the results of domain prediction for MCC1 and MCC2 exhibited variations among the three servers employed. The NIH server predicted the existence of unique SMC domains in certain MCC1 and MCC2 sequences that were investigated. Neither PFAM nor HMM servers identified this domain in the species studied. We investigated this line further by comparing MCC1 sequences against the SMC family sequences identified using the NIH server. However, the Blastp results did not indicate a significant homology between the two groups (MCC sequences and the SMC families). Therefore, we excluded the SMC domain from our subsequent analysis. However, experimental validation could shed light on this controversial aspect.

### 2.2. Ancestral Sequence Reconstruction and Network Split Results

**Figure 2:**
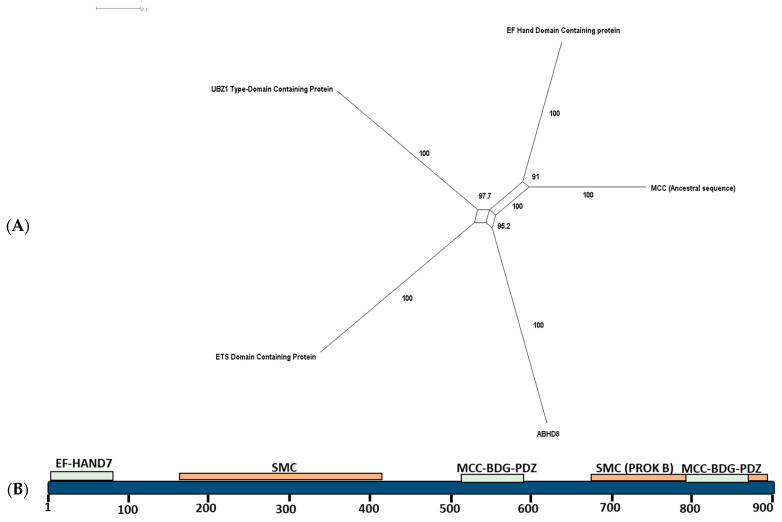
Ancestral sequence reconstruction of the MMC family. (**A**) The nearest homolog for the reconstructed ancestral sequence resembles that of an EF-hand domain-containing protein. (**B**) The EF-hand domain-containing protein structure contains two MCC-PDZ domains, a single EF-hand domain, and an SMC domain.

Our ancestral sequence reconstruction indicates that the MCC’s nearest-most ancient homolog is an EH hand domain-containing protein. We reconstruct the ancestral sequence of the MCC family using MegaX. The sequence reconstruction was based on our generated phylogenetic tree (Figure 1 and Figure 2A). We searched for homologs for the generated ancestral sequence using BLASTP and HMM search servers. The sequences that obtained the highest scores corresponded to an EF-hand domain-containing protein, a UBZ1 type domain-containing protein, an ETS domain-containing protein, and ABHD8. The results and the reconstructed sequence were fed into the SplitsTree program. The results show that the nearest homolog to the ancestral sequence is an EF-hand domain-containing protein (Figure 2A,B).

#### 2.2.1. Sites of Functional Divergence

A study of site-specific shifts in evolutionary rates following gene duplication was performed using Sequence Harmony. Multiple sites seem to play a primary role in the specificity of the divergence between MCC/MCC1 and MCC2. One of the important residues that seems to play a critical role in determining the functional specificity between the two groups is 737-N (Asparagine) (Table 1). All the MCC1 groups have an N residue, while MCC2 has E, D, or R. This residue lies in the first MCC-bdg_PDZ domain of the MCC1 group and hints at the possible role of MCC-bdg_PDZ in the functional divergence between the two groups.

#### 2.2.2. Motifs

We investigated the existence of differential motifs between the MCC1 and MCC2 sequences (Table 2). Several distinctive motifs are found solely in one of the groups. For example, EP-WETQDSF, which is known to play a role in the coatomer construction of the vesicles coat, could be identified in primates in MCC2, but not in MCC1 (Figure 1). Another example is GRHAPPGE. This motif functions as a tankyrase-binding site by interacting with the ankyrin repeat domain region in Tankyrase-1 and Tankyrase-2 (TNKS). TNKS are modulators of the Wnt/β-catenin signaling pathway. Notably, we found the NPSTGE motif in MCC1, but not in MCC2. This motif binds to the Kelch domain of KEAP1 with high affinity. NPSTGE is required for the efficient recruitment of target proteins to the Cul3-based E3 ligase to enable KEAP1 to regulate the function of Nrf2 (NFE2L2).

### 2.3. Positive Selection

We conducted a positive selection analysis for the MCC family. Our results indicate that the MCC family does not seem to have evolved under significant positive selection. Conversely, on several levels of analysis, namely, general, branch, and site, our results indicate that the MCC evolutionary pattern followed a negative selection pattern. (i) For the global model, we used the M0 model to estimate the possibility of positive selection over the whole tree. We found the ω value calculated using PAML for the M0 model to be 0.45728, *p*-Value < 0.00001. (ii) Similarly, for the branch values, for MCC1 vertebrates, the ω value was 0.89, suggesting purifying selection, without strong evidence of statistical significance. Also, in the case of the MCC2 branch, the ω value was 0.64046. On the site level, we did not detect any sites that were subjected to significant positive selection (i.e., ω > 1) (Table 3).

## 3. Discussion

Our study provides evidence supporting the ancient origin of the MCC family. In addition to MCC1 and MCC2 homologs in vertebrates, we identified a single MCC homolog in various invertebrate species (e.g., Trichoplax and Cnidarians) (Figure 1). Selection analysis did not reveal any sites subjected to significant positive selection, indicating overall structure conservation between the two families and within each family. The structure of the MCC family includes two main elements: (i) MCC-PDZ and (ii) the EFh-binding domain. The structure of MCC1 in vertebrates as well as Cnidaria, Spiralia, and Spiders includes two MCC-PDZ domains, while the MCC2 sequence exhibits a single MCC-PDZ domain (Figure 1). All vertebrates’ MCC1 proteins possess an EFh-binding domain, except for Vombatus. We have located an EFh-binding domain in the elephant shark (XP_007895580.2). Similarly, the MCC homolog in Oedothorax gibbosus (Gibbous dwarf spider) contains an EFh-binding domain, while no other invertebrate species investigated contain this particular domain. Our ancestral sequence reconstruction analysis indicates that the MCC family ancestral sequence homolog had at least one MCC-PDZ domain. Our phylogenetic network indicates that the nearest homolog to the ancestral sequence of the MCC could have contained an EFh-domain that was lost in Trichoplax and Cnidaria, but reappeared in Spiders. It is also plausible that the emergence of the EP-hand domain first appeared in Spiders (Figure 2A).

Our results indicate a divergence of function between MCC1 and MCC2. We have found nine motifs conserved in MCC1, but not in the MCC2 family. Additionally, 14 motifs were specific only to MCC2. However, in the case of MCC1, these functional motifs were highly conserved in most of the species investigated. For example, Mod(r) is a motif encompassing 150 residues. It is conserved in various eukaryotic proteins investigated and is homologous to the Drosophila melanogaster Modifier of rudimentary “Mod(r)” proteins [12]. Modr’s primary function is to enable lysosomal sorting [13]. This motif is highly conserved within all MCC1-expressing species. As a result, the Mod (r) proteins may play a primary role in the functional specification between MCC1 and MCC2. However, whether MCC1 could play a role in lysosomal sorting is yet to be determined. The QILGSLPN motif is highly conserved in MCC2. QILGSLPN was shown to play a role in alternative splicing (Table 2) [14,15]. However, whether MCC2 plays a role in alternative splicing is not yet known.

Our research suggests that MCC1 and not MCC2 could be contributing to Treg cells’ function in colorectal cancer. In our recent publication, using microarray analysis of the Th17/Treg pathway, we revealed that several genes that regulate the cell cycle are upregulated in Treg, but not in Th17 [11]. MCC1 was upregulated in Tregs, but not in Th17. Several other genes linked to controlling the cell cycle were also differentially expressed. For example, we found that WWP2, which is known to control the cell cycle, is upregulated in Treg, but not in Th17 [16]. In this current study, we found that the CGRKKSSC (364–371) motif could be contributing to the functional specificity of MCC1. This motif is a known site for fucose residue attachment to serine and is implicated in Notch signaling [17,18]. Notch signaling plays a role in regulating the cell cycle [19]. Importantly, our results indicate that MCC1 and not MCC2 possess the motif NPSTGE. This motif binds to the Kelch domain of KEAP1 with high affinity. KEAP1 is one of the primary regulators of the cell cycle, both dependently and independently of Nrf2 [20,21]. MCC1 has been implicated in inhibiting and enhancing the cell cycle, as its function seems cell-specific. MCC1 inhibits the cell cycle in colorectal epithelial cells, but positively supports B lymphocyte malignancies [9]. Treg cells support colorectal cancer growth by inhibiting Th17 [22]. MCC1 contains an EFh-binding domain which was shown to bind calcium ions. EF-hand domain binding to calcium ions is involved in various functions, such as buffering calcium in the cytosol, signal transduction, and the contraction of fibers in vertebrates and invertebrates [23,24,25]. Treg uses calcium for its differentiation, and the Treg-mediated regulation of conventional T cells involves the suppression of calcium signaling. The calcium inhibition of calcineurin reduces NfkB via an IKK-mediated pathway. Based on the existence of the EFh-binding motif (known to mediate calcium signaling) in MCC1 and not in MCC2, and the upregulated expression of MCC1 in Treg cells, we can speculate on a putative role for MCC1 in Treg calcium regulation, possibly through regulating calcineurin-mediated pathways [26,27]. Thus, MCC support of Treg differentiation through enhancing the cell cycle could be supporting cancer development.

Study limitations: Our study provides valuable insights into the regulatory role of MCC1 in Treg differentiation by utilizing extensive RNA-seq data analysis. However, further experimental studies are warranted to expand upon these findings. In vitro assays could be conducted to investigate the impact of MCC1 overexpression or deletion on Treg differentiation. Additionally, in vivo studies using adoptive transfer in appropriate mouse models, such as Rag1-/-mice, could elucidate the effect of MCC on CD4+ T cell differentiation in the context of colorectal cancer genesis and development. These complementary experimental approaches will not only strengthen the evidence supporting the role of MCC in Treg function, but also provide valuable insights for potential therapeutic interventions targeting MCC in colorectal cancer.

## 4. Methods

### 4.1. Database Search

This study aimed to examine the relationship between the molecular evolution of the MCC family and its functions. We reasoned that due to the diversity of this protein family, studying protein sequences rather than DNA sequences might be more informative. Furthermore, we selected 12 species and genera that span more than 500 million years to ensure that our analysis is a fair representation of the evolutionary history of the MCC family. BLASTP searches were conducted using human MCC1 and MCC2 protein sequences against the proteomes of Chimpanzee (*Pan troglodytes*), House Mouse (*Mus musculus*), Common Wombat (*Vombatus ursinus*), Platypus (*Ornithorhynchus anatinus*), Red Junglefowl (*Gallus gallus*), Zebrafish (*Danio rerio*), Sea Squirt (*Ciona intestinalis*), Arthropoda, Spiralia, Cnidaria, Trichoplax, and Sponge (*Amphimedon queenslandica*) [28]. Whenever one protein possessed more than one transcript, only the longest transcript was used in the analysis [29]. Sequences were selected as candidate proteins if their E values were <1 × e^−10^ [28]. Conserved domains were investigated using the CDD function on the NIH website (accessed on 25 August 2022), PFAM, and HMRR. Based on the consensus domains determined by these three domain-predicting methods, sequences were filtered for the presence of conserved domains homologous to human MCC1 and MCC2 domains, respectively.

### 4.2. Alignment and Phylogenetic Analysis

The phylogenetic investigation was performed in three stages. First, MCC family amino acid sequences were aligned in Seaview using MUSCLE. After that, we employed PhyML to determine the best phylogenetic tree to represent the interrelationships among the MCC family homologs. We used the LG model with empirical values calculated for amino acid equilibrium frequencies [30]. Invariable sites and across-site rate variations were calculated using an optimized algorithm.

### 4.3. Functional Divergence Estimation and Motif Search

We used Sequence Harmony and Multi-RELIEF to identify functional differences within the MCC subfamilies that may contribute to the disparity in function within the Th17/Treg axis. Sequence Harmony compared the two groups of sequences (e.g., MCC/MCC1 and MCC2 families) to identify the variable amino acids and their distribution frequency. The positions, where the amino acid compositions differ between the two groups, were assigned low score values. A score of zero indicates distinct amino acids at that position, while a score of one signifies nearly identical amino acid compositions. Multi-RELIEF predicts the residue specificity of residues by performing two comparisons. The first comparison is between each sequence and its nearest homolog within the same group, and the second comparison involves comparing each sequence with its closest homolog in the second group. A residue is considered specific if it exhibits a high specificity score in at least one of the two comparisons. Additionally, we conducted an extensive motifs search using an ELM server http://elm.eu.org/ accessed on 30 September 2022 with a motif cut-off value of 100. We also conducted a motif search using www.genome.jp accessed on 3 October 2022 [31].

### 4.4. Positive Selection Analysis

We utilized the maximum likelihood approach method to investigate the selection process that governed the MCC family’s evolution. We back-translated the downloaded protein sequences using the EMBOSS server (https://www.ebi.ac.uk/Tools/st/emboss_backtranseq, accessed on 5 September 2022) to estimate the cDNA of the investigated sequences [32]. After that, we used CODEML-PAML (V4.4) to estimate the substitution rate ratio (ω) given by the ratio between nonsynonymous (dN) and synonymous (dS) mutations. We utilized four models: general (basic), branch, branch-site, and sites. The main difference between these models is their level of investigation. While the global model assumes a constant ω ratio for all the trees investigated, the branch model calculates two ω values (the branch investigated and the tree, respectively). The branch-site model calculates ω values for each nucleotide on a specific branch, while the site model estimates ω values for each nucleotide in the alignment. Statistical significance is calculated based on the χ^2^ test [33].

## Figures and Tables

**Table 1:** Functional divergent sites.

Pos	Entropy				SH	Rnk	Consensus	
Ali	A	B	AB	Rel.			A	B
772	1.19	1.84	2.38	1.05	0.00	11	Qne	ATlp
770	1.21	1.84	2.39	1.05	0.00	11	Lefr	DPav
909	0.82	1.56	2.04	1.05	0.00	9	Tav	PLS
891	0.41	1.84	1.89	1.05	0.00	6	Rt	QLav
886	1.21	1.84	2.39	1.05	0.00	6	Kamp	QEgl
949	0.00	1.15	1.37	1.05	0.00	4	D	Rhk
701	0.82	0.86	1.78	1.05	0.00	4	Sct	Gr
948	0.82	1.15	1.89	1.05	0.00	4	Lfv	Pnr
684	1.42	0.59	2.06	1.05	0.00	4	Ivqt	Ph
698	0.82	1.66	2.08	1.05	0.00	4	Rms	Qchk
946	1.21	2.13	2.50	1.05	0.00	4	Qdps	Aegkt
686	1.42	1.84	2.52	1.05	0.00	4	Edkp	SAtv
1162	0.65	0.00	1.36	1.05	0.00	3	Kr	Q
900	0.41	0.99	1.57	1.05	0.00	3	Ki	RQ
1128	0.41	1.15	1.63	1.05	0.00	3	Ed	Rqt
715	0.98	0.59	1.79	1.05	0.00	3	IL	Ag
711	0.81	1.38	1.97	1.05	0.00	3	Dn	Rqe
709	0.82	1.66	2.08	1.05	0.00	3	Qde	Flrv
898	1.21	1.15	2.14	1.05	0.00	3	Qeks	Rhn
901	1.21	1.38	2.22	1.05	0.00	3	Ndks	Eaq
1190	1.42	1.15	2.27	1.05	0.00	3	Lmft	Asv
1126	1.28	1.84	2.44	1.05	0.00	3	SNl	KDat
1189	1.78	1.66	2.69	1.05	0.00	3	Aslnt	Rehq
952	0.41	0.00	1.21	1.05	0.00	2	Nt	K
954	0.00	1.15	1.37	1.05	0.00	2	V	Qae
1004	0.82	0.00	1.47	1.05	0.00	2	Vch	L
1153	1.04	0.00	1.61	1.05	0.00	2	Edk	R
1154	0.41	1.15	1.63	1.05	0.00	2	Kr	Gns
1131	0.82	0.59	1.68	1.05	0.00	2	Hay	Rq
1132	0.41	1.84	1.89	1.05	0.00	2	Qi	ASrv
410	0.82	1.38	1.97	1.05	0.00	2	Edn	Rqh
1092	0.82	1.45	2.00	1.05	0.00	2	Kqr	DEl
876	1.58	1.84	2.63	1.05	0.00	2	Tlmnv	KApr
737	0.00	1.38	1.46	1.05	0.00	1	N	Edr
1142	0.00	1.38	1.46	1.05	0.00	1	K	Cfs
1185	0.00	1.84	1.63	1.05	0.00	1	K	AEst
734	0.41	1.38	1.72	1.05	0.00	1	Yf	Rqh
730	0.00	2.13	1.73	1.05	0.00	1	L	Qekrs

**Table 2:** Motifs analysis and their location.

Motif	Position	Known Function	Species	Family
NPSTGE	496–501 [A]	Motif binds the Kelch domain of KEAP1 with high affinity.	H, C, M, P, V, G, D	MCC1
AGSSS	10–14 [A]	SPOP is part of the complex SPOP/Cul3 and it plays an important role in protein degradation ubiquitination.	H and C	MCC1
LQKLLE ALHKLLT	167–173 [A] 184–190 [A]	LXXLL supports binding to nuclear receptors. RORγt is a well-known nuclear receptor, but it is unknown whether MMC can bind to it.	H, C, M, P, and G	MCC1
CGRK	364–367 [A]	Peptide C-terminal amidation, possibly to protect against degradation.	H, C, M, P, V, G, D	MCC1
NSC	117–119 [A]	NSC is a motif for N-glycosylation, a post-translational modification process where glycans are added to specific Asn residues in proteins in the endoplasmic reticulum and Golgi apparatus.	H and C	MCC1
GGSSLH/P	175–180 [A]	NEK2 is a phosphorylation motif recognized and targeted by protein kinases for phosphorylation.	H, C, M	MCC1
CGRKKSSC	364–371 [A]	This motif is a site for the attachment of a fucose residue to a serine, indicating the possible involvement of fucosylation in the regulation or function of that protein.	H, C, M, P, V, G, D	MCC1
RKKSSCS KKSSCSL	366–372 [A] 367–373 [A]	The motif sequence is recognized by PKA as a specific target for phosphorylation.	H, C, M, P, V, G, D	MCC1
LKSE	857–860 [A]	SUMO-1 may attach to that motif in a Sumoylation process. Sumoylation can have transcriptional regulation, DNA repair, nuclear transport, and protein quality control.	H, C, M, P, V, G	MCC1
GRHP/APPGE	13–20 [A]	The motif may function as a docking site for Tankyrase-1 and 2, regulating protein interactions, localization, and stability.	H, P, V	MCC2
LPPP	693–696 [A]	LPPP motif acts as a docking site in calcineurin substrates to allow them to interact with the catalytic and regulatory subunits of calcineurin and facilitate participation in cellular signaling events.	H, C	MCC2
FAPP	42–45 [A]	This motif is recognized by the EVH1 domains, which are specific protein domains found in the PPP4R3 regulatory subunits of the PP4 holoenzyme.	H	MCC2
MSARA	1–5 [A]	This motif is found in pro-apoptotic proteins and it counteracts caspase inhibition by the Inhibitor of Apoptosis Proteins in apoptotic cells.	H, M, V, P	MCC2
EPWETQDSF	251–259 [A]	This motif mediates the coatomer subunit delta construction. Coatomer plays a part in forming vesicle coat.	H, C, M	MCC2
RAWDPEKLA	583–591 [A]	This short motif is found in cargo proteins and mediates kinesin-1-dependent microtubule transport by binding to the KLC TPR region.	H, C, M	MCC2
PTLAP PPLPP	447–451 [A] 691–695 [A]	PxLxP motif is recognized by a subset of MYND domain-containing proteins. MYND domain is involved in transcriptional regulation, chromatin remodeling, protein localization, and signal transduction	H, C	MCC2
QILGSLPN	276–283 [A]	The PTB RRM2 Interacting (PRI) motif is found in some splicing regulators	H, C	MCC2
PPQLGD	695–700 [A]	TRAF2-binding motif. Members of the tumor necrosis factor receptor (TNFR) superfamily recruit TNFR-associated factors (TRAFs) to initiate cell signaling.	H, C	MCC2
PPLP	691–694 [A]	PPLP is a motif recognized by WW domains of Group II that play a role in multiple cellular processes.	H, C	MCC2
M/LAHPLL	261–266 [A]	Dileucine motifs involved in the trafficking and sorting of proteins in the endosomal-basolateral-lysosomal pathway	H, C, M	MCC2
EAW/SRLL	384–389 [A]	Dileucine motifs involved in the trafficking and sorting of proteins in the endosomal-basolateral-lysosomal pathway	H, C, M	MCC2

**Table 3:** Positive selection models investigated.

Model	ω
general	0.45728, *p*-value is <0.00001
branch	ω value was 0.89 *p*-value > 0.005
branch-site	ω value was 0.64046 *p*-value > 0.005
sites	No sites with ω > 1

## Data Availability

All raw data are available upon request.
